# Relationship between occlusal force and endothelial function in community‐dwelling elderly women: A pilot study

**DOI:** 10.1002/cre2.630

**Published:** 2022-07-22

**Authors:** Kanna Kato, Naoko Matsuda, Miki Takahata, Chika Koseki, Michiyasu Yamaki, Toshiaki Sato

**Affiliations:** ^1^ Area of Occupational Therapy, Graduate School of Health Sciences Yamagata Prefectural University of Health Sciences Yamagata City Yamagata Prefecture Japan; ^2^ Yamagata City Welfare Council Kanai Regional Comprehensive Support Center Yamagata City Yamagata Prefecture Japan; ^3^ Department of Occupational Therapy Yamagata Prefectural University of Health Sciences Yamagata City Yamagata Prefecture Japan

**Keywords:** community‐dwelling, elderly, endothelial function, occlusal force

## Abstract

**Objective:**

In this study, age, endothelial function as flow‐mediated dilation (FMD), occlusal force, grip strength, and advanced glycation end products (AGEs) were obtained. AGEs were measured as indicators of aging, while grip strength was measured as an indicator of muscle strength. This study aimed to explain the relationship between occlusal force and endothelial function and determine whether occlusal force can be a new indicator in community preventative care projects.

**Materials and Methods:**

In 38 community‐dwelling women (age, 76.7 ± 5.7 years), the occlusal force and grip strength were measured, the endothelial function was evaluated by FMD, and AGEs were obtained. The relationship between occlusal force, measurement items, and factors were investigated independently related to endothelial function.

**Results:**

There were significant correlations between occlusal force and grip strength (*r* = .54, *p* < .01). The degree of FMD was significantly associated with occlusal force (*r* = .60, *p* < .01) and grip strength (*r* = .35, *p* < .05) or increase in AGEs (*r* = −.37, *p* < .05). Multiple regression analysis revealed that occlusal force was significantly associated with the degree of FMD (*p* < .01).

**Conclusion:**

Occlusal force can be an important indicator of endothelial function in the community‐dwelling elderly. This study may help understand the general health of the elderly in communities.

## 1 INTRODUCTION

To have a long life in a community, it is important to prolong the healthy life expectancy and the duration of living without requiring any assistance for activities of daily living (ADLs). Accordingly, it is essential to maintain and improve the physical strength and quality of life (QOL) of the elderly through preventative care in a community.

Eating is a crucial part of ADLs; it plays an important role in determining oral function and QOL, especially in the elderly (Ohtani et al., 2002; Rouxel et al., 2017). Previous studies showed that occlusal force is associated with physical function, (Umeki et al., 2018; Yamaguchi et al., 2018, 2019) cognitive function, (Hatta et al., 2019) nutritional status, (Okamoto et al., 2019), and all‐cause mortality in the elderly (Iinuma et al., 2016; Ohi et al., 2019).

Endothelial function is also related to lifestyle habits, (Wang & Widlansky, 2009) such as diet and exercise, (Rossman et al., 2017) and it is assessed by flow‐mediated dilation (FMD). A decreased FMD increases the risk of arteriosclerosis, cerebrovascular disease, and myocardial infarction (Ras et al., 2013; Targonski et al., 2003). These diseases may have residual sequelae even after recovery and can be considered risk factors for shortening the healthy life expectancy. FMD can be improved by reviewing lifestyle habits. An appropriate evaluation and understanding of FMD can provide countermeasures against associated diseases that shorten the healthy life expectancy of the elderly.

Both occlusal force and FMD can be important indicators of the general condition of the elderly and can be used for appropriate preventative care measures in communities. However, to measure FMD, a specialized medical institution must be visited, which is time‐consuming; further, the procedure requires technique. In contrast, measuring the occlusal force is simple and requires no special technique. The measuring equipment is portable; therefore, it can be measured at various places in communities. We considered that if a certain relationship between occlusal force and FMD is found, occlusal force evaluation could be an indicator for the elderly to understand their general condition. However, only a few studies examined the relationship between occlusal force and FMD.

This study aimed to investigate the relationship between occlusal force and FDM and to evaluate advanced glycation end products (AGEs) as an indicator of aging and grip strength as an indicator of muscle strength. Furthermore, it was examined whether occlusal force could be a new indicator for preventative medical services in communities.

## 2 METHODS

### 2.1 Participants

In this study, 38 elderly women (76.7 ± 5.7 years) were analyzed. The participants were aged >65 years during the investigation, living in their own homes, walking independently, and participating in their community salon programs or preventative care more than once a week. The exclusion criteria were those with severe dementia, higher functional disabilities, severe diabetes and heart diseases, severe medical illnesses, functional or organic oral cavity problems, and those who cannot occlude their upper and lower molars (sections), not limited to their teeth.

First, community salons in each area were visited to explain the outline of our research and to request participation in this study. Following this, a dental hygienist conducted a survey wherein the numbers of teeth of individuals who agreed to participate in the study were determined (primary survey). Furthermore, participants who agreed to participate in a secondary survey underwent a second survey, and parameters other than the number of teeth were measured at the Yamagata Prefectural University of Health Sciences.

After the primary survey of 71 respondents, a small number of male subjects were excluded. Furthermore, the secondary survey excluded 27 subjects because they refused to participate, could not be contacted, and so forth; therefore, the total number of subjects in this study was 38.

### 2.2 Variables

#### 2.2.1 Endothelial function

**Figure 1:**
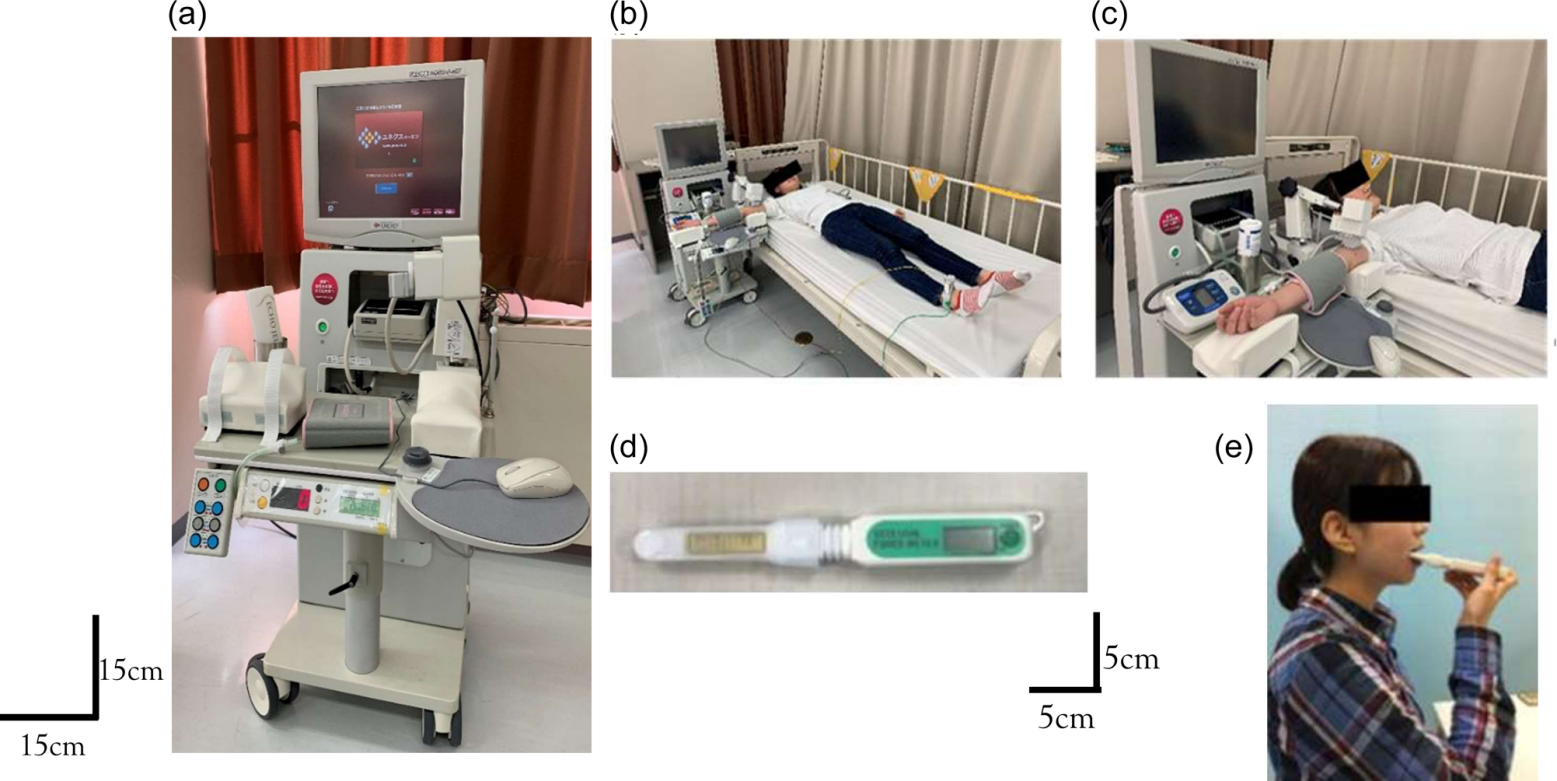
Measuring tool and position. (a–c) Measuring tool and position for flow‐mediated dilation. (d, e) Measuring tool and position for occlusal force.

FMD was measured as an endothelial function using an instrument equipped with software for monitoring the brachial artery diameter (UNEX38G; Unex Co. Ltd., Japan) (Figure 1a). Measurements were taken in 15–20 min. The subjects were placed in a supine position with a blood pressure cuff placed around their forearm (Figure 1b,c). The brachial artery was scanned longitudinally 5–10 cm above the elbow by an ultrasonic echo. When the clearest B‐mode image of the anterior and posterior intimal interfaces between the lumen and vessel wall was obtained, both tracking gates were placed on the intima, the artery diameter was automatically tracked, and the waveform of diameter changes over a cardiac cycle was displayed in real time using the FMD mode of the tracking system. After measuring the blood pressure, the blood pressure cuff was inflated to 50 mmHg above the systolic pressure for 5 min and released. Blood vessel diameter changes were measured for 2 min after the 5 min inflation. The baseline diameter was defined by measuring the minimum blood vessel diameter for 20 s after releasing the blood pressure cuff. FMD was automatically calculated as a percentage change in peak vessel diameter from the baseline value. The reference value was 7% or more. Endothelial dysfunction was suspected in less than 4% (Maruhashi et al., 2019). Figure 1a–c shows the measuring tool and subjects' position.

#### 2.2.2 Occlusal force

The occlusal force was measured using an occlusal force meter (GM10; Nagano Keiki, Japan) (Figure 1a,b). Measurements were taken twice on the right and left sides and the larger value was considered the final value. The subjects were instructed to bite as hard as possible to take measurements during contraction (Kato et al., 2020).

#### 2.2.3 AGEs

An increasing level of AGEs was thought to affect aging. Thus, as indicators of aging, these were measured using an AGE Reader (AGE Reader SU 4; Dignoptics, Netherlands) (Haus et al., 2007; Semba et al., 2010). The subjects were seated with their forearm on the AGE Reader in a prone position (Meerwaldt et al., 2004). Three measurements were on the same side of the forearm and the average value was considered the final value.

#### 2.2.4 Grip strength

Grip strength was measured using a Jamar hand dynamometer (SH‐5001; SAKAI Med, Japan). The subjects were seated with their elbow joint flexed at 90° and both their feet shoulder‐width apart. Measurements of the dominant hand were taken twice and the largest value was considered in the prone position (Meerwaldt et al., 2004) as the final value.

### 2.3 Statistical analysis

The normality of the data was confirmed using the Shapiro–Wilk test. Correlations between parameters were assessed by bivariate simple correlation analysis. For age, occlusal force, grip strength, FMD, and AGEs, spearman's rank correlation coefficient was used for analysis. Multiple regression analysis was performed on the degree of FMD as a dependent variable and occlusal force, grip strength, AGEs, and age as independent variables. IBM SPSS version 24 (IBM Japan, Tokyo, Japan) was used for all analyses, and *p* < .05 was considered statistically significant.

### 2.4 Ethics approval and consent from participants

This study was approved by the Institutional Review Board of Yamagata Prefectural University of Health Sciences (Approval number: 1906‐06). Oral explanations were provided to the participants and written consent was obtained before initiating the study.

## 3 RESULTS

The total number of subjects in this study was 38 and their characteristics are shown in Table 1. The mean age of the subjects was 76.5 ± 5.7. Table 2 shows their grip strength, occlusal force, AGEs, and FMD. Table 3 shows the correlation coefficient for age, occlusal force, grip strength, FMD, and AGEs. There was a significant correlation between occlusal force and grip strength (*r* = .54 and *p* < .01). The degree of FMD significantly was associated with occlusal force (*r* = .60 and *p* < .01) and grip strength (*r* = .35 and *p* < .05) or amount of increased AGEs (*r* = −.37 and *p* < .05). Table 4 shows the result of multiple regression analysis with the degree of FMD as a dependent variable. The independent variables were age, AGEs, grip strength, and occlusal force. The occlusal force (*p* < .01) was identified as a significant factor. The *R* was .61, and the adjusted *R* ^2^ was .3. The standard partial regression coefficient was 0.59 in occlusal force.

**Table 1:** Characteristics of participants

*N*			38
Age (years)			76.7 ± 5.7
Height (cm)			150.8 ± 5.4
Body mass index (kg/m^2^)			24.0 ± 4.0
The number of remaining teeth (*n*)			19.9 ± 7.7

**Table 2:** Characteristics of parameters measured in participants

	Median	IQR	Minimum	Maximum
Grip strength (kg)	22.5	6.3	8.0	32.0
Occlusal force (kN)	149.5	232.3	0.0	650.0
AGEs (AU)	2.2	0.5	1.3	3.2
FMD (%)	7.1	2.1	2.6	10.4

Abbreviations: AGE, advanced glycation end products; FMD, flow‐mediated dilation; IQR, interquartile range.

**Table 3:** Correlation matrix of parameters

	Age	Occlusal force	Grip strength	AGEs	FMD
Age (years)	1	−.07	−.35*	.23	.03
Occlusal force (kN)		1	.54**	−.36*	.60**
Grip strength (kg)			1	−.26	.35*
AGEs (AU)				1	−.37*
FMD (%)					1

Abbreviations: AGE, advanced glycation end products; FMD, flow‐mediated dilation.

* *p* < .05

** *p* < .01.

**Table 4:** Multiple regression analysis values with degree of FMD as the dependent variable

	Standard partial regression coefficient	*p* Value	95% CI		*R*	Adjusted *R* ^2^
			Lower	Upper		
Age (years)	0.14	.32	−0.05	0.14	.61	.30
Occlusal force (kN)	0.59	<.01	0.00	0.01		
Grip strength (kg)	0.01	.96	−0.12	0.12		
AGEs (AU)	−0.08	.62	−1.66	1.00		

Abbreviations: AGE, advanced glycation end products; CI, confidence interval; FMD, flow‐mediated dilation.

## 4 DISCUSSION

In this study, the relationships among oral function, FMD, and AGEs were investigated. We think that occlusal forces, FMD, and AGEs can be useful in understanding the general condition of the elderly in preventative care. This is the first study to investigate the relationship between occlusal force and FMD. Many previous studies have focused on occlusal force and grip strength (Hatta et al., 2019; Umeki et al., 2018; Yamaguchi et al., 2018). In this study, a relationship was found between occlusal force and grip strength, which was consistent with the results reported in previous studies.

AGEs were measured as indicators of aging. Previous studies showed that AGEs accumulate with age (Haus et al., 2007; Semba et al., 2010). However, in the present study, no specific relationship between AGEs and age was found. However, their detailed life background was not examined. In the future, we may be able to prove the relationship by conducting a survey that includes the subjects' life backgrounds.

The results of this study indicate a relationship between occlusal force and FDM. To the best of our knowledge, only a few studies investigated the relationship between occlusal force and endothelial function. This is a novel finding. From a functional and structural point of view, endothelial dysfunction may result in a reduced blood flow through the microcirculation, which may lead to muscle fiber atrophy. A previous study reported a strong correlation between blood flow volume changes and muscle protein synthesis changes (Rasmussen et al., 2006). Furthermore, endothelial dysfunction also leads to a decreased secretion of vascular endothelial growth factor (VEGF), facilitating functional muscle ischemia; moreover, a decreased secretion of VEGF in association with endothelial dysfunction may lead to a negative muscle protein balance (Zempo et al., 2017).

Residents need to understand their health condition and live consciously in preventative care. Therefore, a simple indicator is necessary. Both occlusal force and FMD are useful for understanding the general condition of the elderly. However, to measure the FMD, a specialized medical institution must be visited, which is time‐consuming. Occlusal force measurement can be easily performed without requiring difficult techniques or time for measurement; its measuring equipment is portable and it can be measured weekly during community salon visits. Multiple regression analysis showed that only occlusal force was independently associated with the presence of endothelial function. This study suggests that occlusal force can be a good indicator of the general condition, physical and mental function, and nutritional status of the elderly; this can help determine early countermeasures against high‐risk diseases that shorten the healthy life expectancy.

This study has a few limitations. First, its sample size was small and limited to a healthy older adult population. The actual situation of male elderly people was also not ascertained. In the future, we would like to collect and analyze data from multiple populations. Second, the possible effects of medications and existing diseases on the outcomes could not be ruled out. Third, this study was designed as a cross‐sectional study and could not evaluate the causal relationship between the reduced occlusal forces and decreased endothelial function. Further research is needed to overcome these limitations.

## 5 CONCLUSION

In conclusion, in this study, a relationship was found between occlusal force and endothelial function in community‐dwelling elderly women. Measuring the occlusal force is easier than measuring FMD. Therefore, the occlusal force can be an important indicator of the general condition and high‐risk diseases that shorten the healthy life expectancy of the elderly.

## AUTHOR CONTRIBUTIONS

**Kanna Kato**: study concept and design, acquisition of subjects and/or data, analysis and interpretation of data, and preparation of the manuscript. **Matsuda Naoko**: acquisition of subjects and/or data, analysis and interpretation of data, and preparation of the manuscript. **Miki Takahata**: acquisition of subjects and/or data, analysis and interpretation of data, and preparation of the manuscript. **Chika Koseki**: acquisition of subjects and/or data and preparation of the manuscript. **Michiyasu Yamaki**: study concept and design and preparation of the manuscript. **Toshiaki Sato**: study concept and design, acquisition of subjects and/or data, and analysis.

## CONFLICT OF INTEREST

The authors declare no conflict of interest.

## Data Availability

The data that support the findings of this study are available from the corresponding author upon reasonable request.
